# Integrating microsystems with metamaterials towards metadevices

**DOI:** 10.1038/s41378-018-0042-1

**Published:** 2019-01-28

**Authors:** Xiaoguang Zhao, Guangwu Duan, Aobo Li, Chunxu Chen, Xin Zhang

**Affiliations:** Department of Mechanical Engineering, Boston University, Boston, MA USA

**Keywords:** Micro-optics, Optical materials and structures

## Abstract

Electromagnetic metamaterials, which are a major type of artificially engineered materials, have boosted the development of optical and photonic devices due to their unprecedented and controllable effective properties, including electric permittivity and magnetic permeability. Metamaterials consist of arrays of subwavelength unit cells, which are also known as meta-atoms. Importantly, the effective properties of metamaterials are mainly determined by the geometry of the constituting subwavelength unit cells rather than their chemical composition, enabling versatile designs of their electromagnetic properties. Recent research has mainly focused on reconfigurable, tunable, and nonlinear metamaterials towards the development of metamaterial devices, namely, metadevices, via integrating actuation mechanisms and quantum materials with meta-atoms. Microelectromechanical systems (MEMS), or microsystems, provide powerful platforms for the manipulation of the effective properties of metamaterials and the integration of abundant functions with metamaterials. In this review, we will introduce the fundamentals of metamaterials, approaches to integrate MEMS with metamaterials, functional metadevices from the synergy, and outlooks for metamaterial-enabled photonic devices.

## Optics: Dynamically tuning how materials respond to light

Specially engineered materials known as metamaterials have led to a boost in the performance of optical and photonic devices, with yet further improvements within reach by finding ways to dynamically tune their properties. Metamaterials consist of arrays of structures smaller than the wavelength of light, offering an unprecedented opportunity to tune their interaction with electromagnetic waves. Xin Zhang’s team at Boston University has reviewed approaches to integrate microelectromechanical systems and nanoelectromechanical systems (MEMS and NEMS) with metamaterials to create metadevices. By using MEMS and NEMS to adjust how the array in a metamaterial is arranged, metadevices can be rapidly and reversibly reconfigured to detect, modulate, or respond to specific electromagnetic frequencies. The authors conclude by highlighting avenues for the improvement of metadevices, which have applications from LIDAR to 5G communication.

## Introduction

Electromagnetic (EM) metamaterials represent an important class of artificial materials composed of arrays of subwavelength unit-cell structures, which are also known as meta-atoms, with engineered effective optical properties, such as effective permittivity and permeability. The responses of the metamaterials mainly depend on the structural design of the unit cells rather than their chemical composition, giving rise to flexibilities in designing their effective optical properties across the entire EM spectrum from low to high frequencies, including microwave to terahertz, infrared, and visible frequency ranges^1–4^. Starting with the experimental realization of negative index materials (or left-handed materials)^5^, which existed only in theory for a long time^6^, metamaterials have enabled numerous appealing applications, including invisibility cloaking^7^, superlensing^8^, and perfect absorption^9^, due to their unprecedented properties. To further enhance the functionality of such metamaterials, current research is increasingly focusing on tunable, reconfigurable, nonlinear, and sensing metamaterials and shifting from fundamental research to practical applications, which is boosting the development of metamaterial devices or metadevices^10^. In metadevices, metamaterials exhibit dynamic properties, enabling the modulation of the intensity and phase of light and the manipulation of near-field interactions and nonlinear responses. Compared to tunable optical devices constructed with naturally available materials, metadevices exhibit higher tunability, more degrees of freedom, and more compact dimensions.

The development of micro- and nanofabrication techniques is essential for the emergence of terahertz, infrared, and visible metamaterials. The feature sizes of terahertz and mid-infrared metamaterials are in the range of a few to tens of micrometers, which lies in the capability of microfabrication based on photolithography^2^. Near-infrared and visible metamaterials require feature sizes of tens to hundreds of nanometers, which can be fabricated using nanofabrication techniques based on electron beam lithography (EBL) and focused ion beam (FIB) milling^11,12^. Furthermore, micro and nano electromechanical systems (MEMS and NEMS) also play important roles in constructing tunable metamaterial devices and generating nonlinear responses, as will be introduced below.

The idea of dynamically tunable metamaterials was developed in the early stage of metamaterials and was first demonstrated at terahertz frequencies^13^. In this design, split ring resonators (SRRs) were patterned on a high-resistivity GaAs substrate. The dynamic control of the electrical response of the SRRs was achieved via photoexcitation of the free carriers to ~4 × 10^16^ cm^−3^ in the substrate. Following this work, tunable magnetic responses^14^, chirality^15^, absorbance^16^, and beam steering^17^ enabled by optical pumps were demonstrated by photodoping the materials in the vicinity of the metamaterials, including semiconductors and varactors. Electrical tuning mechanisms were also implemented by electrically gating the constituting materials, such as semiconducting materials^18–20^ and graphene^21^, to modulate the collective response of metamaterials. Moreover, liquid crystals^22,23^ and phase change materials, such as the chalcogenide glass^24^, the vanadium dioxide^25–27^ and superconductors^28–30^, have also been incorporated into metamaterials to generate tunable responses.

In addition to changing the properties of the materials that make up metamaterials, structurally reconfiguring meta-atoms is another efficient approach to tune the metamaterial response^31–34^. Compared to tunable metamaterials enabled by their material properties, mechanically tunable metamaterials are more stable and much easier to tune by changing individual meta-atoms, and they are capable of achieving larger tunability and dynamic ranges, especially broader frequency tuning ranges. The mechanical approach allows the control of EM waves in highly compact metamaterial devices. However, integrating metamaterials with MEMS and NEMS requires sophisticated design of the system layout to achieve the desired function, and the fabrication process must be well designed. In this review, the integration of metamaterials with MEMS and NEMS, which can cause mechanical deformation in metamaterials and in turn modulate their effective properties, will be introduced. In the next section, the basic principles of metamaterials and the actuation mechanisms of MEMS and NEMS are introduced. In the “Applications” section, a variety of applications enabled by combining MEMS/NEMS and metamaterials are introduced, including frequency and amplitude modulation, polarization conversion, wave front control, tunable absorption and emission, and EM detection and nonlinear devices. In the “Outlook” section, the future directions of mechanically tunable metamaterials are discussed.

## Metamaterials and MEMS/NEMS

Metamaterials are assemblies of subwavelength unit cells, i.e., meta-atoms, that can give rise to effective EM properties, including electric permittivity (*ε*_eff_) and magnetic permeability (*μ*_eff_), and they are analogs to natural materials consisting of atoms. When an incident EM wave impinges upon a metamaterial, a resonant response in the meta-atoms is excited, leading to Lorentz-type dispersion in *ε*_eff_ and/or *μ*_eff_^35^, which may be extracted from the simulated or experimental transmission and reflection spectra^36^. Of note, the meta-atoms’ size and spacing are much smaller than the working wavelengths of the EM waves; therefore, the diffraction effects are suppressed. Different meta-atom geometries can be designed to achieve the desired properties of the metamaterials, including permittivity^37^, permeability^11^, anisotropy^38^, and chirality^39^. By controlling the resonant properties of the meta-atoms, one may tune the effective properties of the metamaterial^32^.

The principle of metamaterials originates from the magnetism in microstructures using nonmagnetic conducting materials^40^. When conducting materials are formed into subwavelength structures, such as Swiss rolls or SRRs, these structures can generate loop currents under the excitation of the magnetic component of an external incident EM field, therefore achieving effective magnetic susceptibility and permeability (*μ*_eff_). Metamaterials provide an efficient way to attain the desired magnetic property. For instance, *μ* = 1 holds for natural materials in the optical regime; however, metamaterials allow us to control the magnetic response from the microwave^40^ and terahertz^2^ to optical regimes^41,42^.

**Fig. 1:**
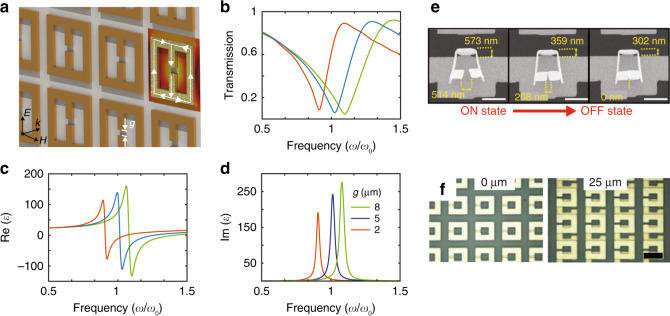
An overview of a typical EM metamaterial design, and approaches of integrating MEMS and NEMS with metamaterials. **a**–**d** A typical metamaterial design consisting of electric split ring resonators (ESRRs) and its response. **a** The resonant current flow direction and electric field distribution are illustrated on one pixel. **b** Simulated transmission spectra of the metamaterial in (**a**) with different gap sizes (**g**). The resonant frequency corresponds to the dip in the transmission spectrum. From the simulated spectrum, the real (**c**) and imaginary (**d**) parts of the effective permittivity can be extracted. The simulation results reveal large tunability in the effective permittivity via structural reconfiguration. **e** Reconfiguring a metamaterial by thermally modifying the gap of SRRs^50^. **f** Structurally modulating the metamaterials by changing the coupling between two layers of the meta-atoms^52^. The scale bars in (**e**) and (**f**) are 1 μm and 40 μm, respectively. **e** Reprinted with permission from ref. 50. Copyright 2016 American Chemical Society. **f** Reprinted with permission from ref. 52. Copyright 2011 by the American Physical Society

The effective permittivity of metamaterials arises from the electric response of meta-atoms. The electric field component of an external incident EM wave excites electric-dipole-like resonating charges in the meta-atoms. Similar to plasmonic resonance, one can achieve effective permittivity through this process. A cut-wire is a basic type of meta-atom to generate effective electric responses^43^. With the increasing demands on compact meta-atoms, symmetric structures, such as electric split ring resonators (ESRRs) (Fig. 1a), have been proposed to generate a resonant electrical response while suppressing the magnetic response^37^.

The anisotropic response of metamaterials originates in the lack of axial symmetry in the meta-atoms, which generates different responses for excitations with different polarizations. For example, “I”-shaped resonators exhibit different responses to waves polarized in the horizontal and vertical directions, which is used for polarization conversion^38^. The chirality of metamaterials stems from the “handedness” of the meta-atoms, which respond to different incident circular polarizations. Chiral metamaterials, in which the electric and magnetic responses are coupled, are an alternative approach to achieving a negative refractive index without enforcing simultaneous negative permittivity and permeability^44^. Helical resonators^39^, three-dimensional chiral meta-atoms^45^, and multiple-layer planar resonator without any symmetry^46^ have been used to design the desired effective indices and optical activity of metamaterials. The relationship between the metamaterial geometry and effective properties has been reviewed comprehensively elsewhere^47^. In addition to metallic metamaterials, dielectric materials with high permittivities were designed to achieve effective optical properties via the electric and magnetic resonance modes^48^.

One commonly used modality of meta-atoms is the ESRR, as shown in Fig. 1a. Under electric excitation (i.e., the electric field is perpendicular to the gap of the ESRRs), an oscillating current is induced in the unit cells, leading to a resonant response in the transmission spectrum as shown in Fig. 1b. One may obtain a Lorentz-like complex effective permittivity (i.e., *ε*; Fig. 1c, d) by fitting the spectra^49^. When the structural properties of the unit cells, such as the size of the gaps, are changed, the effective permittivity is modified significantly, which demonstrates that one can achieve metamaterials with significant tunability by changing the geometry of their unit cells. Micro/nano-mechanical actuators are the enabling methods for controlling the structural properties of unit cells. Figure 1e shows an example of employing thermal micro-actuators to manipulate the gap of an SRR, which achieved significant amplitude modulation in the transmission response at infrared frequencies^50^.

In addition to structural modification, intra-meta-atom and inter-meta-atom near-field coupling cause another degree of freedom that may be used to modulate the metamaterial response. Broadside coupled split ring resonators (BC-SRRs) are one type of metamaterials consisting of meta-atoms that include a pair of strongly coupled SRRs, which are vertically separated by 180° relative rotation to each other^51^. By changing the relative position between the two resonators in one unit cell, one can efficiently modulate the effective response of the metamaterial^52,53^, as shown in Fig. 1f. This metamaterial design allows efficient coupling between the materials’ EM properties and the mechanical displacements of the unit cell components, enabling control of the propagation of light and, more generally, EM waves.

**Fig. 2:**
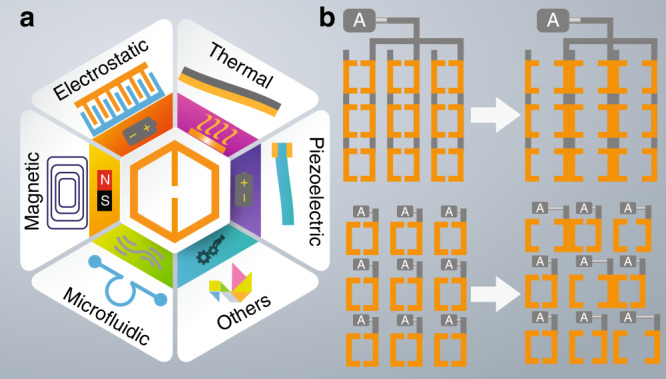
An overview of actuation mechanisms in MEMS/NEMS-based metamaterials. **a** Different actuation mechanisms, including electrostatic, thermal, piezoelectric, and magnetic actuators, microfluidic channels and other structural designs (e.g., origami), can interact with meta-atoms to produce metamaterials with nontrivial tunability. **b** The MEMS/NEMS actuators can be integrated in the metamaterials to drive the overall meta-atom array (top) or individual meta-atoms (bottom). In (**b**), “A” represents the actuator

Incorporating MEMS/NEMS techniques in metamaterial designs enables the manipulation of the structural configurations of metamaterial unit cells in real time. Since the emergence of MEMS/NEMS, this technique has shown exceptional performance in driving and sensing micro- and nanoscale mechanical displacements in confined spaces, making it suitable for constructing structurally reconfigurable metamaterials. Different actuation mechanisms can be incorporated with metamaterials to tune their effective properties as listed in Fig. 2a.

Electrostatic actuation is broadly employed in MEMS due to its inherent advantages, including fast response, low power consumption, large travel distance, and ease of fabrication. Electrostatic actuators can accurately generate in-plane and out-of-plane linear displacements as well as rotations, enabling applications such as gyroscopes^54–56^, RF switches^57^, micromirrors and scanners^58–60^, microtweezers^61^, and micropositioners^62^. Essentially, the driving force of an electrostatic actuator stems from the Coulomb force between charges. If a pair of electrodes is placed near each other and has a voltage difference, positive and negative charges will accumulate on the electrodes. If one of the electrodes is fixed, and the other is movable, the accumulated charges will create an attraction force between the plates and cause a displacement of the movable plate. The force exerted by an electrostatic actuator is determined by the applied voltage and the electrode configuration, including the overlapping section area and the separation distance. The displacement of the movable electrode is related to the electrostatic force and the compliance of the supporting structures. To achieve large displacements at low driving voltages, electrodes with high voltage-force conversion efficiencies, such as comb-drive electrodes, are used to generate the force. In addition, the structure to support the movable electrodes must be designed with a lower stiffness^63^. In general, the travel range of electrostatic actuators is usually hundreds of nanometers to tens of microns, which is suitable for constructing tunable metamaterials working at terahertz, infrared, and visible frequencies. Relatively large electrostatic actuators, such as comb-drive actuators, can actuate the overall array of meta-atoms, whereas compact actuators, such as cantilevers, may be integrated into individual subwavelength meta-atoms to reconfigure the metamaterial.

Thermal actuation utilizes the differences in the thermal expansion coefficients of materials to generate mechanical displacements with temperature variations. One widely used configuration is the bimorph thermal actuator. This type of actuator comprises two layers of materials with different thermal expansion coefficients and generates out-of-plane displacements^64^ when their temperature changes. A single-layer material, which is usually silicon, is patterned to specific shapes (such as a U-shape or chevron beams) and can cause large in-plane displacements under thermal excitation^65,66^. One can drive thermal actuators using either an increase in external temperature or internal Joule heating in active regions of the structure. Thermal actuation is a good candidate to achieve large forces (on the order of a few mN) and displacements (up to tens of μm) with low input voltages. However, its drawback is the relatively low response speed and high power consumption. Thermal actuators can be integrated with metamaterials to actuate either the overall metamaterial or control individual unit cells to modulate their effective properties. Recently, Au/SiN_*x*_ bimaterial thermal actuators have been included in asymmetric SRRs to modulate the infrared reflection of metamaterials^67^. Several studies have shown that metamaterials can also absorb photon energy and convert it to thermal energy, which in turn can drive thermal actuators, serving as EM wave detectors.

In addition to electrostatic and thermal actuation methods, many other actuation mechanisms, including magnetic and piezoelectric actuation, also have significant potential in metamaterial applications. For example, magnetic actuation is based on the Lorentz force experienced by a current-conducting wire in an external magnetic field. This actuation introduces control over the metamaterial response via the magnetic field and is an analog to the magneto-optical effect. For example, chevron gold nanowire array patterned silicon nitride thin films were designed as plasmonic metamaterials and served as conductors for magnetic actuation currents^68^. By applying a bias of several volts, the gold nanowires are driven by the Lorentz force in an external magnetic field. The deformation of the gold nanowires induces reversible metamaterial transmission responses, which demonstrates a magneto-optical effect. In addition to generating magneto-optical effects, the magnetic actuators can be integrated in metamaterial unit cells to achieve tunable and nonlinear responses. For example, the magnetic force can deform helical resonators under an EM excitation, which modulates the resonance response of the metamaterial^69^. Piezoelectric actuation is another approach to drive optical metamaterials using the converse piezoelectric effect, which actuates mechanical displacements at the scale of micrometers with a driving voltage of a few volts^70^. Piezoelectric actuators can be integrated into metallic metamaterial unit cells, such as SRRs, to control the unit cell geometry, which in turn modulates the EM response^71^. Piezoelectric materials can serve as the unit cell to achieve controllable permeability^72^ and tune the response of the resonators^73^. They have been employed to construct tunable waveguides and prisms for wave manipulation^74^. More intriguingly, the mechanical resonance of piezoelectric actuators is sensitive to temperature variations; therefore, they can be integrated with metamaterial absorbers to form EM wave detectors^75^. In addition to solid-state actuators and sensors, microfluidic devices can also be used to construct tunable metamaterials^76,77^. In this type of device, microfluidic channels, which are patterned as arrays, can carry liquid metals (such as mercury) instead of water-based solutions to form metamaterials. By controlling the shape of the liquid metal using pneumatic valves, one can tailor the metamaterials’ responses^78^. Due to the limitations of liquid metal properties, most microfluidic tunable metamaterials operate at microwave and terahertz frequencies. In addition, metamaterial unit cells floating in a liquid environment can mimic liquid crystals, in which the orientation of meta-atoms can be controlled by external electric fields to induce tunable EM responses at terahertz frequencies^79^. Recently, the emergence of new structural designs, such as origami, provides an efficient way to achieve mechanically reconfigurable metamaterials with high tunability^80,81^.

In addition to enabling the designs of effective properties, metamaterials are capable of controlling topologies, i.e., Chern numbers, in the photonic dispersion bands in the reciprocal space, enabling photonic topological devices^82^. Similar to the topological properties of condensed matter, photonic topological insulators support unidirectional spin-polarized light propagation at the interface^83^. By changing the shape of the metamaterial unit cells, the Chern number of the topological photonic structures can be altered to manipulate the propagation of EM waves. For example, metallic rods with collars sandwiched between two metal plates have been used to construct a microwave metamaterial with hexagonal periodicity^84^. The locations of the collars can modify the inversion symmetry of the metamaterial and therefore tailor the Chern number of the topological metamaterials. Integrating MEMS/NEMS actuators with topological insulators enables functional devices to manipulate wave propagation^85^.

By combining MEMS actuators with metamaterials, the electromagnetics, mechanics, electronics, thermal dynamics, and fluidic dynamics work in synergy to create tunable and reconfigurable metamaterials, which are functional metadevices for applications such as EM wave modulation and sensing. Compared with conventional optical devices, metadevices are design-driven and have unprecedented advantages, including enhanced performance, a large dynamic tuning range, design flexibility, fast response, and compactness. The integration schemes between metamaterial and MEMS/NEMS actuators can be classified into two types. The first is to actuate the overall meta-atom array to tune the collective response, as shown in the top row of Fig. 2b. The second integration scheme is to pair an actuator with each meta-atom. The challenge of integrating metamaterials with MEMS/NEMS actuators lies in the meta-atom design, material selection, and fabrication. The organic integration of metamaterials and MEMS/NEMS actuators can be achieved by standard surface and/or bulk micromachining techniques, and specific examples are given in the following examples.

## Applications

Combining MEMS/NEMS design and EM metamaterials can be used in various applications including the modulation and sensing of EM waves. It has been reported that one can achieve the modulation of the resonant frequency, polarization, wave front, and power of EM waves. Furthermore, by utilizing the absorption properties of some metamaterial designs, one can take advantage of the MEMS components’ thermal deformation properties to detect EM waves. In the following section, we further discuss the application of MEMS/NEMS-based EM metamaterials for wave modulation and sensing.

### Modulation of frequency and amplitude

One of the major functions of MEMS/NEMS in tunable metamaterials is to modulate the metamaterials' performance. The tuning of the amplitude and resonant frequencies of the transmission or reflection, for example, is frequently examined in the literature. Some devices are also capable of rapid modulation of such properties. For example, some early designs of MEMS-based tunable metamaterials used a discrete micromachined RF switch to tune the resonant frequency of a meta-atom at microwave frequencies^86^.

**Fig. 3:**
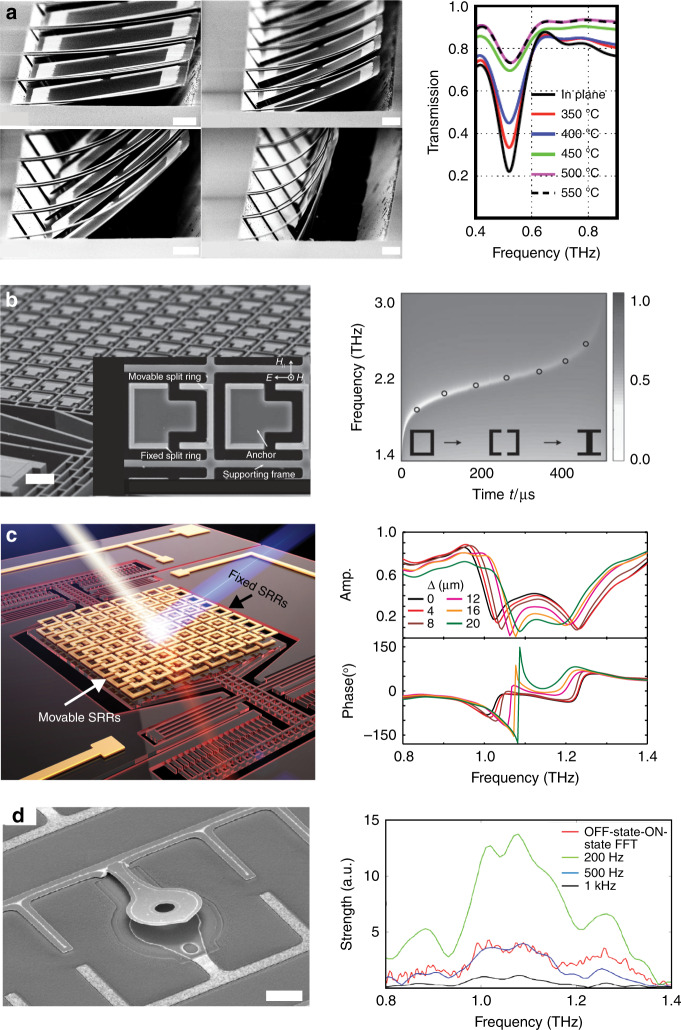
Modulators based on MEMS metamaterials. **a** The first MEMS tunable metamaterials enabled by thermal actuators for amplitude modulation^87^. **b** MEMS-actuated single-layer metamaterials for resonant frequency modulation^92^. **c** A dual-layer tunable metamaterial enabled by micromachined comb-drive actuators and its reconfigurable resonant response^105^. **d** A THz modulator operating at a modulation frequency of 40 kHz based on MEMS cantilever actuators^88^. The scale bars in (**a**), (**b**), and (**d**) are 10 μm. In (**c**), the unit cell of the metamaterial is 40 μm. **a** Reprinted with permission from ref. 87. Copyright 2009 by the American Physical Society. **b** Reprinted with permission from ref. 92. Copyright 2011 by John Wiley and Sons. **c** Reprinted with permission from ref. 105. Copyright 2016 by Springer Nature. **d** Reprinted with permission from ref. 108. Copyright 2014 by the Optical Society

As an example of using MEMS for EM wave amplitude modulation, a mechanically reconfigurable metamaterial at terahertz frequencies was implemented by integrating planar arrays of SRRs with arrays of bimaterial thermal actuators^87^, as shown in Fig. 3a. Under external thermal excitation, the orientation of the SRRs with respect to the incident EM radiation was altered, thereby modulating the transmission amplitude of the metamaterial. Subsequently, bimaterial actuators were employed to thermally control the inter-meta-atom coupling, leading to a profound modulation of the metamaterial’s transmission response at infrared frequencies^88^. These early thermally reconfigurable metamaterials were driven by changes in the environmental temperature. To control the metamaterial more actively, localized resistive heating was employed to drive the thermal actuators to modulate the transmission amplitude^50,89,90^. Integrating phase-transition materials with MEMS actuators to generate more degrees of freedom in controlling the metamaterial response provided a multifunctional micro-electro-opto-mechanical system (MEOMS) platform towards metadevices^91^.

Modulating the resonant frequency of metamaterials is another intriguing application of MEMS actuators. Changing crucial geometries of meta-atoms is efficient for modulating metamaterial resonant responses. Figure 3b shows a tunable metamaterial with its unit cells shaped into pairs of split rings with a “[]” shape. In this design, one ring in each unit cell was fixed, whereas the other was mounted on a supporting frame actuated by comb-drive electrostatic actuators^92^. By controlling the voltage applied to the comb-drive, the gap between the split rings was adjusted to modulate the resonant frequency of the metamaterial. The change in the resonance was achieved within 0.5 ms, demonstrating a high modulation speed. Other meta-atom geometries, such as asymmetric SRRs^93^ and Maltese crosses^94^, have also been employed in single-layer MEMS-actuated metamaterials to obtain a tunable resonant response. In addition to MEMS actuators, compliant and stretchable substrates offer another option to structurally modify the structure of meta-atoms, thereby tuning the metamaterial resonant frequencies^95,96^. The displacement induced by the stretching process was normally up to 1 μm, making it more suitable for efficient tuning at infrared and visible frequencies^97–99^. Some early stretchable metamaterials were actuated using customized test jigs, which were not sufficiently compact. Recently, MEMS actuators and patterned polymer scaffolds were integrated to stretch the meta-atoms, achieving tunable responses^100^.

In addition to modifying the meta-atom geometry, changing the coupling between meta-atoms is another efficient way to modulate the resonant responses of metamaterials^51,52^. MEMS actuators can drive the relative displacement between coupled meta-atoms by actively and dynamically adjusting the coupling coefficient between meta-atoms with single-layer^101–104^ or multilayer^105^ coupled resonators. In a previous study, dual-layer broadside coupled SRRs were integrated with comb-drive actuators to form one stationary layer of SRRs and one movable array of SRRs. The actuators can induce the lateral displacement of the movable SRR array layer to change the interlayer inductive and capacitive coupling and modulate the resonant response of the metamaterial^105^, as shown in Fig. 3c. When the two arrays aligned well (i.e., Δ = 0 μm), the two layers of SRRs were strongly coupled and exhibited two resonant frequencies due to mode splitting. The lower frequency corresponded to the symmetric mode, whereas the higher frequency corresponded to the anti-symmetric mode. With the lateral shift of the movable layer, the symmetric mode redshifted and the anti-symmetric mode blueshifted as the coupling weakened. The resonant frequencies shifted by approximately 40 GHz when the displacement approached its limit (~25 μm) with an actuation voltage of 80 V. Along with the resonant frequency modulation, the transmission amplitude was modulated by 11 dB at 1.03 THz, and the phase modulation range was ~180° with a response time of 1.3 ms. These experimental results, which are supported by theoretical analysis and numerical simulations, revealed the potential of applying MEMS-enabled dynamically coupled metamaterials in chemical sensing, communication, and EM wave manipulations.

MEMS actuators can provide steady-state or low-frequency modulation and further provide high-frequency modulation due to the nature of MEMS structures’ mechanical resonances. The mechanical resonant frequency of a MEMS structure is related to the effective stiffness and mass of the resonator. Higher stiffnesses and smaller masses correspond to higher resonant frequencies^106^. Cantilevers, which are an important class of MEMS resonators for RF applications, are suitable for constructing harmonically tunable metamaterials due to their high mechanical resonant frequencies^107^. In a metamaterial-based terahertz modulator, as shown in Fig. 3d, MEMS cantilevers were integrated in ESRR meta-atoms to achieve a modulation depth of 16.5 dB at 480 GHz with a driving voltage of 40 V^108^. Modulation frequencies up to 1.5 kHz were experimentally measured using THz time-domain spectroscopy, which limits the frequency range of dynamic characterization. A 40 kHz modulation frequency, which corresponds to the mechanical resonant frequency of the cantilevers, was expected for a continuous wave (CW) THz source. Similarly, fixed–fixed beam MEMS actuators with higher stiffness were employed to modulate the metamaterial response at 200 kHz^109^. When cantilever resonators are miniaturized to the nanometer scale, they can form tunable metamaterials that can modulate infrared radiation at 32 MHz^110^. Such NEMS-based metamaterials usually operate at infrared frequencies and exhibit higher modulation frequencies than MEMS resonators’ working frequencies, which mainly fall in the terahertz or microwave range.

Using MEMS and NEMS actuators, one can structurally reconfigure meta-atoms to modulate their resonant frequency, amplitude, and phase in steady and dynamic fashions. For different applications, meta-atom designs and actuation mechanisms may be optimized to maximize the resonant frequency shift, amplitude modulation depth or phase coverage^111,112^. Specific examples will be discussed in the following sections.

### Controllable polarization conversion

Polarization control of EM waves is of great interest for modern optical applications. Traditionally, birefringent materials are employed to implement alternating polarization. The operating spectrum is usually limited by the material response. Moreover, the devices usually suffer from bulky size and a narrow working frequency bandwidth due to the requirement that the waves have to propagate along the material to accumulate phase retardance^113,114^. To address these limitations, metasurfaces with designed unit-cell structures were recently reported to perform polarization manipulation^38,115^. The phase response of metasurfaces stems from the resonance of these engineered structures and therefore enables a compact design with subwavelength thickness^10^. More importantly, the operating spectrum can be arbitrarily tuned from the microwave to optical regime by scaling the feature size of the unit cells. However, the operational bandwidth of such metasurfaces is usually narrow, and the operating frequency is fixed after the implementation of the devices^38^. To overcome these limitations, active tuning is used so the metasurfaces can target broader operational frequency ranges. This type of reconfigurable polarizer is more suitable for practical applications.

**Fig. 4:**
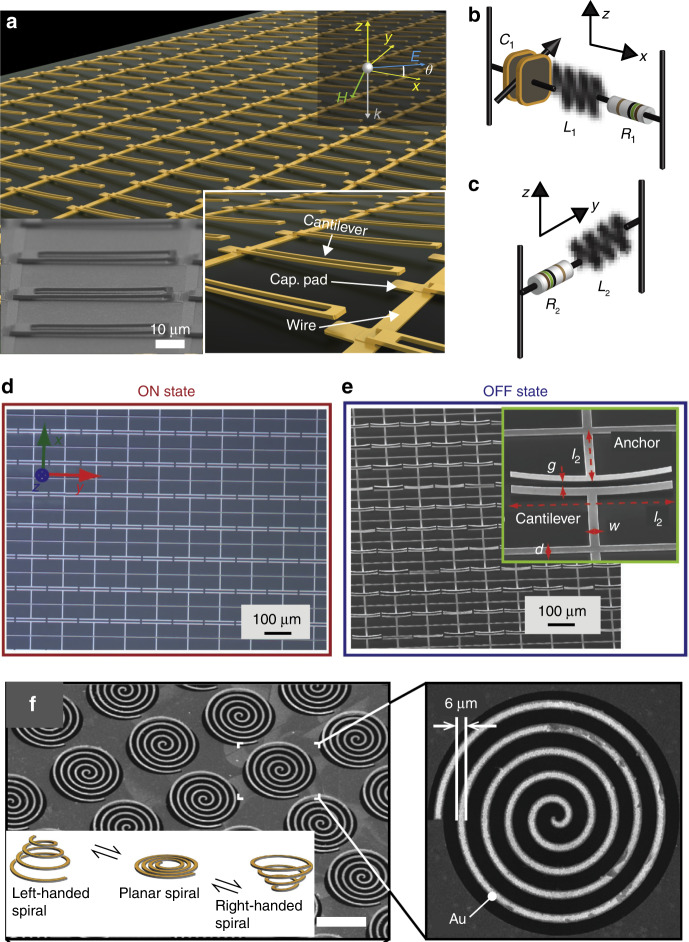
MEMS metamaterials for polorization manipulation. **a** Schematic of a metasurface with a cantilever array working in transmission. The insets show an SEM image of the fabricated sample, a schematic of a single cantilever in cross-section, and a zoomed-in image of the metasurface^116^. **b**, **c** Lump circuit models for incident waves polarized along the *x* and *y* axes. **d**, **e** SEM images of the metasurface with the cantilever array working in reflection in the on and off states^117^. The insets in (**d**) and (**e**) show schematics of a single cantilever in cross-section and a zoomed-in SEM image of a cantilever unit cell in the off state, respectively. **f** SEM image of a metasurface with spiral unit cells. The inset shows the chirality responses with different deformation directions^118^. The scale bar in (**f**) is 100 μm. **a**–**c** Reprinted with permission from ref. 116. Copyright 2018 by the Optical Society. **d**, **e** Reprinted with permission from ref. 117. Copyright 2016 by John Wiley and Sons. **f** Reprinted with permission from ref. 118. Copyright 2015 by Springer Nature

Microcantilevers can again be employed as unit cells to make up birefringent metasurfaces that are dynamically tunable by applying electrostatic forces. As shown in Fig. 4a, the cantilever array was fabricated on a slightly doped silicon substrate coated with a silicon nitride insulation layer^116^. Figures 4b, c schematically illustrate the origin of the birefringent response resulting from the well-separated resonant frequencies of the cantilever array along the *x* and *y* axes. The cantilever tip and the capacitor pad form a tunable capacitor, illustrated in Fig. 4b as C_1_, which can be tuned by applying a voltage between the silicon substrate and the cantilever array. The tunable response along the *x* axis with a fixed response along the *y* axis enables the dynamic control of the polarization of the transmitted radiation. With a right-handed polarized incident wave, the device can dynamically control the polarization of the transmitted waves to achieve linear or left-handed polarized waves. In addition, the tunable cantilever metasurface can also work in a reflection configuration with more than 300° phase tunability^117^. The inset in Fig. 4e schematically shows cantilevers with a three-layer configuration. The ground plane ensures that the device works in reflection mode, and the bimorph configuration of the cantilever (Al_2_O_3_ with Al) creates the initial structural bending during the releasing process for voltage tunability.

Spiral unit cells can also be utilized to rotate linearly polarized incident waves into an arbitrary polarization direction or to elliptically polarize waves by introducing nonzero off-diagonal terms in the Jones matrix^118^. The spiral array was implemented with an SOI wafer by employing deep reactive ion etching. Figure 4f shows a scanning electron microscope (SEM) image of the fabricated spiral unit cells. Large vertical deformation of the unit cells can be controlled with an electrostatic force^119^ or pneumatic force^118^. The handedness of the transmitted wave depends on the deformation direction of the unit cells as illustrated in the inset in Fig. 4f. A polarization rotation of up to ±28° was experimentally achieved, which demonstrated that this design had the potential to act as a practical and compact polarization convertor.

In addition to the tuning schemes presented above, comb drive-based MEMS structures and fluidic channel-based tuning mechanisms have also been used to change the polarization of EM waves. With a comb drive design, unit cell arrays were patterned on both fixed and movable frames. The responses along the *x* and *y* axes can be controlled by shifting the relative position of the unit cells on the comb drive platform^94,120^. In another example, liquid metal was employed to form a unit cell with tunable structures on a fluidic channel platform. The geometries of the liquid metal unit cells could be actively tuned through microfluidic manipulations, realizing multiple functionalities, such as linear-to-linear, linear-to-circular, and linear-to-elliptical polarization conversion in the microwave regime^121^.

### Wave front manipulation

Negative index metamaterials can form super lenses to focus EM waves to break the diffraction limit^8,122^. Recently, metasurfaces have been reported to control the wave front of light using subwavelength micro/nanostructures, which has demonstrated considerable potential in optical applications with reduced thickness and weight to replace conventional bulky optical devices^123,124^. Metasurfaces can introduce a phase discontinuity along the interface to steer the refracted beam following the generalized Snell’s law of refraction^125^

1 $$\sin \left( {\theta _t} \right)n_t - \sin \left( {\theta _i} \right)n_i = \frac{{\lambda _0}}{{2\pi }}\frac{{d\phi }}{{dx}}$$

where *θ*_*t*_ is the angle of refraction, *θ*_*i*_ is the incident angle, *n*_*t*_ and *n*_*t*_ are the refractive indices of the two media on the incident and refractive sides, respectively, *λ*_0_ is the vacuum wavelength, and *dϕ*/*dx* is the gradient of the phase discontinuity along the interface. Equation (1) implies that the refracted beam can have an arbitrary direction with a suitable *dϕ*/*dx*. Similarly, the reflected beam direction can also be calculated with the generalized Snell’s law^126^. Moreover, metasurfaces can be employed as metalenses to focus the incident light. When the phase shift *φ*(*x*,*y*) introduced by the metasurface at (*x*, *y*) is of the form^127^

2 $$\varphi \left( {x,\,y} \right) = - \frac{{2\pi }}{{\lambda _0}}\left( {\sqrt {x^2 + y^2 + f^2} - f} \right)$$

it can compensate for the phase difference between the refracted radiation originating at that point and at the zero-order refraction point, thereby enabling all the refracted radiation to be focused at the focal point at distance *f*.

**Fig. 5:**
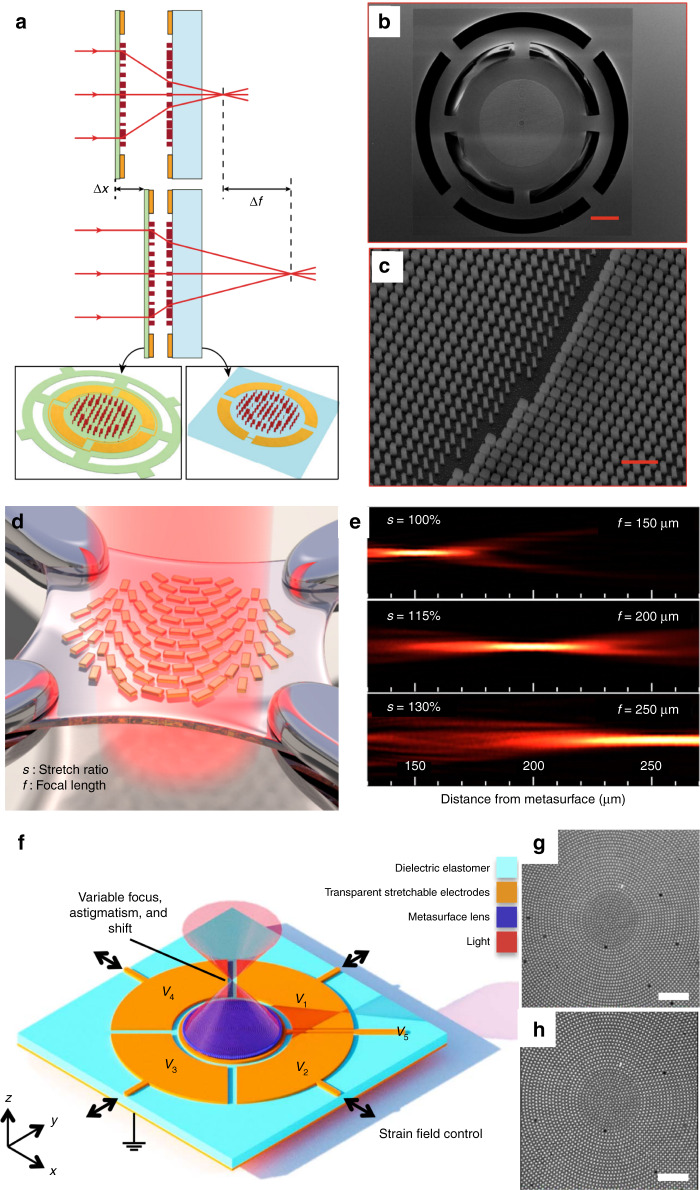
MEMS metamaterials for wave front manipulation. **a** Schematic of the tuning of the focal length of a dual-layer metalens. **b**, **c** SEM images of the top view of the fabricated metalens and a zoomed-in view of the metasurface^128^ (scale bars, 100 μm in (**b**), 1 μm in (**c**)). **d** Schematic of a stretchable metalens. **e** Focal length tuning with different stretch ratios^131^. **f** Schematic of a voltage-tunable metalens. **g**, **h** Optical microscope images of the metalens (scale bars, 20 μm) with no voltage and with 2.5 kV applied to the center electrode, respectively^133^. **a**–**c** Reprinted with permission from ref. 128. Copyright 2018 by Springer Nature. **d**, **e** Reprinted with permission from ref. 131. Copyright 2016 by American Chemical Society. **f**–**h** Reprinted with permission from ref. 133. Copyright 2018 by American Association for the Advancement of Science

MEMS platforms have been introduced to dynamically tune the focal length with dual layer metasurfaces^128^, as illustrated by the schematic in Fig. 5a. The metasurfaces are composed of amorphous silicon posts with a square cross-section. The phase shift is determined by the side length of the posts^129,130^. The first metasurface is fabricated on a silicon nitride membrane with a focal length of approximately 120 μm, and the second is patterned on a glass substrate with a focal length of approximately −130 μm (the minus sign denotes the defocusing). Electrodes are designed around the metasurfaces to change the distance between the two metasurfaces with an electrostatic force, so the focal length can be tuned accordingly. With a 1 μm change in the distance, the metalens experimentally demonstrated more than a 60 μm shift in the focal length.

Metasurfaces can also be fabricated on a stretchable substrate. By mechanically stretching the substrate, the space between the unit cells is changed to tune the focal length by changing the phase discontinuities^131^. As illustrated in Fig. 5d, gold nanorods are embedded inside a polydimethylsiloxane (PDMS) substrate. Circularly polarized incident radiation is scattered into two parts: waves of the same polarization as that of the incident radiation and waves of the opposite circular polarization with phase discontinuities determined by the orientation of the nanorods^132^. By arranging the phase discontinuities according to Equations (1) and (2), the functionalities of beam steering and focusing were realized, and the tunability of the focal length was demonstrated experimentally by stretching the substrate as shown in Fig. 5e. The deformation of the soft substrate can also be achieved by employing dielectric elastomer actuators^133^. The metasurface was composed of amorphous silicon posts embedded in an acrylate elastomer membrane with electrodes attached on both sides as shown in Fig. 5f. A strain field generated by an electrostatic force can compress the membrane to control the space between the nanoposts, resulting in focal length tuning. From a focal length of 50 mm, the fabricated device exhibited up to 107% focal length modulation (103.5 mm).

Microfluidic channels are another promising approach to achieve local phase discontinuity tuning^134,135^. SRRs with different orientations and gap sizes can scatter polarized incident waves into cross-polarized waves with different phase shifts covering a 2*π* range^136^. To experimentally implement the tunable split rings, a microfluidic network was designed with a pneumatic system to individually control the filling factor of liquid metal and air inside a ring-structured channel with different orientations. The fabricated device can dynamically tune the wave front to realize different focal lengths and steering directions at operational frequencies in the microwave regime. Moreover, with recent advances in micro/nanofluidics, the concept could be transferred to higher frequencies, including the optical range. Zhao et al. creatively demonstrated the use of a plasmonic fluidic lens to achieve tunable focusing of surface plasmon polaritons (SPPs) in the near-infrared regime^137^.

### Tunable absorption and emission

Metamaterial absorbers, which form one of the most flourishing branches of metamaterials, has attracted tremendous research interest and efforts due to their reduced thickness (compared to the operational wavelength) and configurable operational frequencies. Metamaterial absorbers are typically configured with a three-layer design, such as metal–insulator–metal with one or both of the metal layers being a metamaterial^9,138^. The absorption peak frequencies are usually measured after the fabrication of the devices. However, dynamic tuning of the response in real time can offer considerable advantages and enable state-of-the-art devices.

**Fig. 6:**
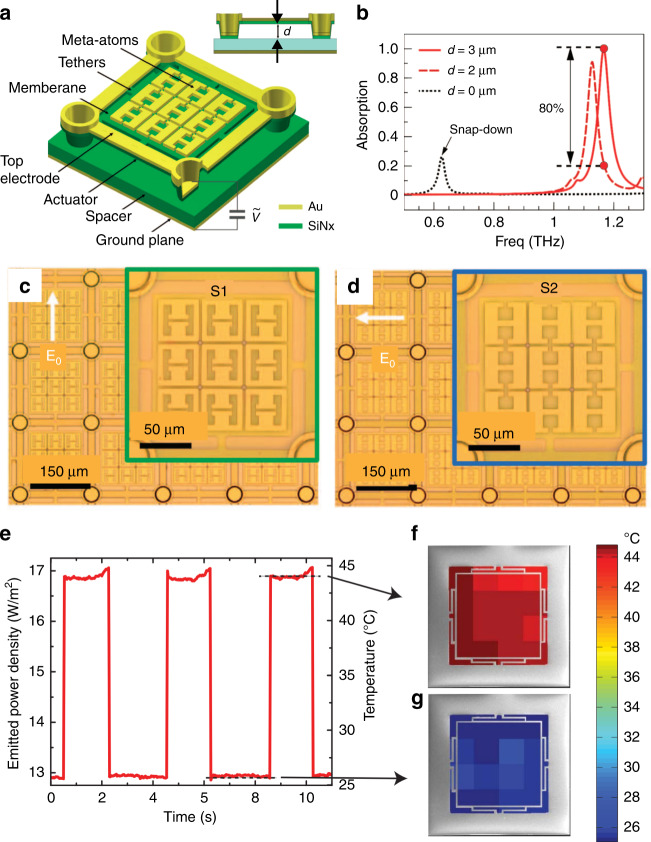
MEMS-based metamaterial absorbers and emitters. **a** Schematic of a voltage-tunable metamaterial absorber^141^. **b** Simulated absorption spectra with different separation distances. **c**, **d** Microscopy images of fabricated tunable metamaterial absorbers with different unit cell geometries. **e** Time dependence of the emitted power density (left *y* axis) and temperature (right *y* axis) in the absorption on and off states. **f**, **g** The spatial dependences of temperature over the sample surface in the on and off states^144^. **a**–**d** Reprinted with permission from ref. 141. Copyright 2017 by Springer Nature. **e**–**g** Reprinted with permission from ref. 144. Copyright 2017 by the Optical Society

MEMS has the advantages of CMOS compatibility, fast response, and a large tuning range. These can be employed to implement tunable metamaterial absorbers^139–141^. Figure 6a shows a schematic of a voltage-tunable metamaterial absorber consisting of a 200-nm-thick metallic ground plane, a 2 μm silicon nitride spacer, and a metasurface supported by a silicon nitride membrane separated from the spacer by a distance of 3 μm^141^. By applying a voltage between the membrane and the ground plane, the electrostatic force can attract the membrane towards the ground plane to change the separation distance, resulting in a modification of the absorption spectrum as shown in Fig. 6b. In addition, metamaterial absorbers can work as metamaterial emitters according to Kirchhoff’s law of thermal radiation^142^. Therefore, by tuning the absorption, the emissions of the metamaterial absorber can be modified to change the emitted power without a change in temperature^143,144^. Assuming a temperature-independent emissivity of the metamaterial absorber, an equivalent temperature change of nearly 20 °C can be generated by switching the metamaterial absorber on and off as shown in Fig. 6e–g.

### EM wave detection and nonlinear response

**Fig. 7:**
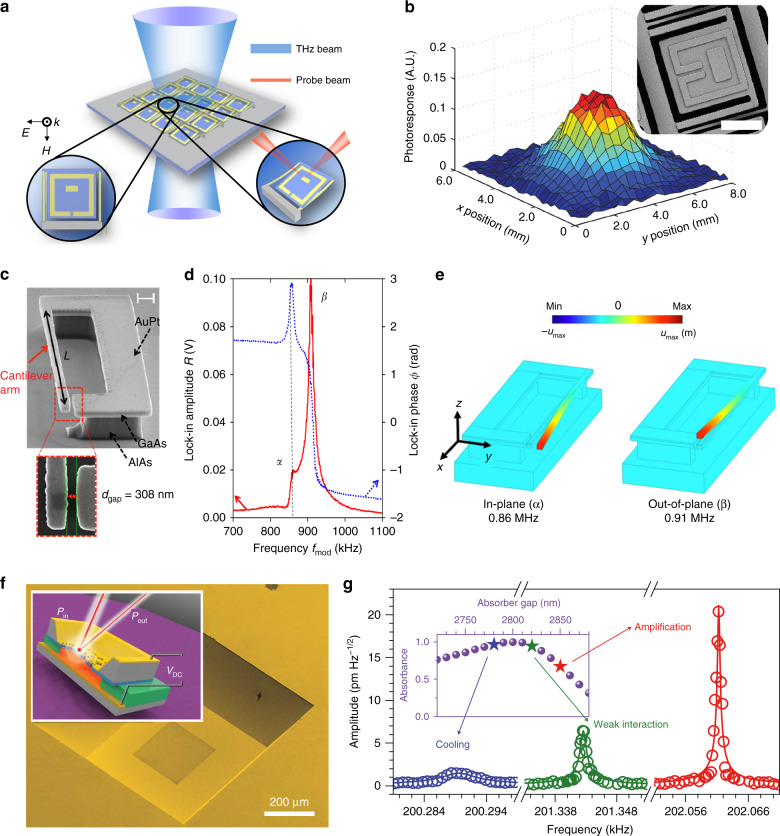
EM wave detection with MEMS metamaterials. **a** Schematic of a metamaterial-enabled focal plane array. The SRRs absorb the incident radiation and convert it to heat, leading to deflection of the bimaterial cantilever, which is probed by an optical laser beam^147^. **b** Profile of a terahertz beam captured by the focal plane array shown in (**a**). Inset: scanning electron microscope image of one pixel in the focal plane array; the scale bar is 20 μm. **c** SEM image of a meta-atom of a metamaterial for terahertz detection using photothermal and Coulomb forces^152^. The scale bar is 1 μm. **d** Experimental amplitude and phase responses of the mechanical vibration versus modulation frequency of terahertz radiation. The resonance peaks (*α* and *β*) correspond to the resonant modes shown in (**e**). **f** A nonlinear metamaterial utilizing the opto-thermal effect to control the dampening effect of the mechanical resonator^154^. **g** Thermomechanical Brownian noise spectra for the fundamental mechanical resonance measured at a pump power of ~17 μW for different absorber gaps, demonstrating the control over the dampening factor. The inset shows the metamaterial absorbance as a function of the absorber gap at a wavelength of 1550 nm. **a**, **b** Reprinted with permission from ref. 147. Copyright 2011 by the Optical Society. **c**–**e** Reprinted with permission from ref. 152. Copyright 2018 by Springer Nature. **f**, **g** Reprinted with permission from ref. 154. Copyright 2016 by Springer Nature

In addition to achieving tunable and reconfigurable metamaterials, the integration of MEMS with metamaterials can lead to high-performance detectors, especially for infrared and terahertz radiation, which has lower photon energies. Using only MEMS techniques, the photothermal effect of microcantilevers was explored to construct uncooled infrared detectors with high responsivity^145^. However, conventional uncooled MEMS radiation detectors suffer from limited absorption and a lack of spectral selectivity, which may limit their applications. Adding metamaterials to uncooled microcantilevers is an efficient way to maximize the absorption coefficient and achieve spectral selectivity^146^. The first integration scheme of a metamaterials-MEMS thermal detector was demonstrated at terahertz frequencies^147^, as shown in Figs. 7a, b. SRR arrays were patterned on the detection pixels supported by bimaterial cantilevers. The SRRs interact with the incident terahertz radiation at resonance and convert it to heat, which leads to the bending of the bimaterial cantilevers. By probing the bending displacement of each pixel, an image of a terahertz pulse can be captured, as shown in Fig. 7b. The responsivity of the detector was measured to be 16,500 V/W with a noise equivalent power of 10^−8^ W/Hz^1/2^. In later studies, different metamaterial perfect absorber configurations and detection approaches have been integrated to form focal plane arrays at terahertz and infrared frequencies^148–151^. Recently, a plasmonic metamaterials-enhanced MEMS photothermal switch was investigated to achieve digitalized infrared detection with near-zero power consumption, providing a new way to construct sensors and detectors^148^.

The interaction between EM waves and metamaterials leads to not only thermal effects but also near-field electric effects, which can also be used for EM wave detection. For example, asymmetric SRRs with a suspended MEMS cantilever were designed and fabricated with gold-coated GaAs thin films as meta-atoms to form the metamaterial^152^, as shown in Fig. 7c, which exhibited an LC resonance mode at 2.7 THz. A THz quantum cascade laser (QCL) operating at 2.7 THz was employed to excite the meta-atoms. When the QCL output was modulated at a frequency (*f*_mod_), mechanical resonance modes could be identified by sweeping the modulation frequency (Fig. 7d). According to the simulation results, the first frequency (860 kHz) corresponded to the in-plane vibration mode, and the second corresponded to the out-of-plane mode (Fig. 7e). Analyses of the amplitude and phase of the mechanical resonance revealed that photothermal and Coulomb forces were explicitly confirmed to cause the THz-induced mechanical vibration. In this detection scheme, the noise equivalent power is estimated to be 0.4 nW/Hz^1/2^, which is similar to that of Golay cells.

Furthermore, the coupling between photonics and mechanics mediated by metamaterials can lead to rich nonlinear physical processes. Specifically, the incident EM energy deforms the metamaterials structurally and in turn changes the metamaterials’ EM response, enabling an intensity-dependent metamaterial response. Plasmonic metamaterials patterned on dielectric nanoscale actuators were demonstrated to have significant nonlinearity due to MEMS actuation^104,153^. The nonlinear effect also opens a new path to optically manipulate mechanical oscillators^154^. As shown in Fig. 7f, gold plasmonic metamaterials patterned on silicon nitride thin films were bonded on a gold ground plane with a 3-μm air gap to form a near-infrared perfect absorber. When a laser is shined on the device, the absorbed energy deforms the membrane and changes the absorbance. By detuning the resonance frequency of the absorber, the light-induced force can either amplify or dampen the mechanical resonance, as shown in Fig. 7g. This phenomenon is analogous to an optical laser or coherent absorber. In addition to the photothermal effect, magnetic forces between broadside coupled SRRs were used to generate changes in their separation distance, leading to nonlinear magnetoelastic metamaterials^155^. Nonlinear MEMS/NEMS metamaterials may inspire new designs for nonlinear responses to construct optical and mechanical devices.

### Summary

Metamaterials have been employed to construct devices to manipulate EM waves via tunable, reconfigurable, and nonlinear effective optical properties enabled by MEMS and NEMS. A variety of applications, including high-speed modulation, tunable polarization conversion, dynamic wave front control, tunable absorption, sensors and detectors, have been demonstrated.

## Outlook

Future efforts in MEMS/NEMS metamaterial research could be directed towards granting access to control the responses of each individual meta-atom via system-level design, forming randomly accessible metamaterials (RAMMs), to achieve on-demand optical responses of the metamaterials. This requires novel meta-atom designs with enhanced tunability, robust actuation mechanisms, reliable system integration, and packaging processes. The realization of RAMMs allows dynamic beamforming to achieve multiple functions, such as dynamic beam focusing and steering, holographic imaging, and orbital angular momentum generation, in a single device. Such devices may play important roles in various applications, such as light detection and ranging (LiDAR)^156^ and 5G communication^157^. Most current MEMS/NEMS actuators in tunable metamaterials operate in an open loop, which may induce errors and instability in actuation. The integration of feedback control in the actuators may be another direction worth pursuing to achieve “smart” and stable reconfigurable metamaterials. Furthermore, structurally tunable metamaterials are efficient at manipulating the near-field interactions in the vicinity of meta-atoms, which allows us to realize nonlinear metamaterials for high harmonic generation and gain-enhancement^158^. In summary, functional metadevices with multiple functionalities are promising and deliverable by the integration of metamaterials with MEMS/NEMS for different applications.
